# Expression of inducible nitric oxide synthase in macrophages inversely correlates with parasitism of lymphoid tissues in dogs with visceral leishmaniasis

**DOI:** 10.1186/s13028-014-0057-z

**Published:** 2014-09-07

**Authors:** Françoise P Sanches, Thaise Y Tomokane, Vânia LR Da Matta, Mary Marcondes, Carlos EP Corbett, Márcia D Laurenti

**Affiliations:** Laboratório de Patologia de Moléstias Infecciosas, Departamento de Patologia, Faculdade de Medicina, Universidade de São Paulo, Av. Dr. Arnaldo, 455-1 andar-sala 1209, CEP: 01246-903 Cerqueira César São Paulo (SP), Brazil; Departamento de Clínica, Cirúrgia, e Reprodução Animal, Faculdade de Medicina Veterinária, Universidade Estadual Paulista, Araçatuba, SP Brazil

**Keywords:** Visceral leishmaniasis, Canine, Nitric oxide, Immunohistochemistry, Infection control

## Abstract

**Background:**

There are only a few studies reporting the role of nitric oxide metabolites for controlling macrophage intracellular parasitism, and these are controversial. Therefore, the present study aimed to evaluate the expression of inducible nitric oxide synthase (iNOS) in the lymph nodes and spleen of dogs affected by visceral leishmaniasis through immunohistochemistry and to determine its correlation with tissue parasite burden and serum interferon (IFN)-γ levels. Twenty-eight dogs were selected and assigned to one of two groups, symptomatic (n = 18) and asymptomatic (n = 10), according to clinical status and laboratory evaluation. A negative control group (n = 6) from a non-endemic region for visceral leishmaniasis was included as well.

**Results:**

Parasite density (amastigotes/mm^2^) was similar between clinical groups in the lymph nodes (*P* = 0.2401) and spleen (*P* = 0.8869). The density of iNOS^+^ cells was higher in infected dogs compared to controls (*P* < 0.05), without a significant difference in lymph node (*P* = 0.3257) and spleen (*P* = 0.5940) densities between symptomatic and asymptomatic dogs. A positive correlation was found between the number of iNOS^+^ cells in lymph nodes and interferon-γ levels (r = 0.3776; *P* = 0.0303), and there was a negative correlation between parasites and iNOS^+^ cell densities both in lymph nodes (r = −0.5341; *P* = 0.0034) and spleen (r = −0.4669; *P* = 0.0329).

**Conclusion:**

The negative correlation observed between tissue parasitism and the expression of iNOS may be a reflection of NO acting on the control of parasites.

## Background

American visceral leishmaniasis (VL) is a zoonotic disease caused by *Leishmania (Leishmania) infantum chagasi* protozoa, which is mainly transmitted by *Lutzomyia longipalpis* sand flies in Latin America [1]. The domestic dog has long been implicated as the main reservoir of *L. (L.) infantum chagasi* due to its close relationship with humans. In areas where zoonotic VL is endemic, the prevalence rate of infected dogs is often high, and it is estimated that more than 50% of seropositive animals identified in field surveys are asymptomatic [2].

Following transmission, parasites initially multiply in skin macrophages at the inoculation site. After localized cutaneous infection, the parasites disseminate via lymphatic or blood vessels, and infect organs rich in mononuclear phagocytes, such as lymph nodes, spleen, liver, and bone marrow [3]. Parasite spreading and infection establishment lead to clinical manifestations, which include lymph node enlargement, hepatosplenomegaly, apathy, decreased appetite, loss of body weight, onychogryphosis, ocular lesions, and skin lesions – such as exfoliative dermatitis, alopecia, as well as pustular, nodular, and ulcerative dermatitis [4].

Protective immunity has been associated with cellular immune response, manifested by delayed-type hypersensitivity (DTH) and a strong proliferative response of peripheral blood mononuclear cells (PBMC) to *Leishmania* antigens [5,6] accompanied by interferon (IFN)-γ and tumor necrosis factor (TNF)-α production. TNF-α is required for macrophage activation and the production of nitrogen-reactive species that are capable of killing intracellular amastigotes [7]. In addition, in vitro experiments have shown that macrophages from dogs infected with *L. infantum* activated with recombinant human (r) IFN-γ produce increased expression of inducible nitric oxide synthase (iNOS), and control parasitism in their cytoplasm [8]. It has also been shown that the high expression of iNOS in macrophages is related with a low number of amastigotes in the skin, lymph node, and liver macrophages of dogs naturally infected by VL [9], but not in the spleen, where the expression of iNOS has been associated with clinical worsening and with high parasitism [10]. Interestingly, resistance to nitric oxide (NO) of some *L. (L.) infantum chagasi* strains isolated from dogs and humans showed parasite survival within the macrophages and a high parasite load, even after macrophage activation by IFN-γ [11].

There are only a few, controversial reports on the role of NO in canine (C) VL; thus, this study aimed to evaluate the expression of iNOS in the lymph nodes and spleens of dogs affected by VL and to correlate the degree of tissue parasitism with iNOS expression in these tissues, and also with IFN-γ levels in serum.

## Methods

### Canine population

The canine population was chosen among dogs destined for euthanasia due to sanitary practices at the Center for Zoonosis Control from the municipality of Araçatuba, São Paulo State, Brazil – a high VL endemic area. Twenty-eight stray mongrel dogs of different ages and genders with positive lymph node smear and immunoglobulin (Ig)G-enzyme-linked immunosorbent assay (ELISA) were enrolled in this study. The animals were divided into one of two groups, symptomatic (n = 18) and asymptomatic (n = 10), according to clinical and laboratory evaluation. After diagnosis, the animals were first anesthetized with sodium pentobarbital (25 mg/kg; Fontoveter, Campinas, Brazil) and blood was obtained by cardiac puncture for IFN-γ serum quantification. According to the Brazilian program for VL control implemented by the Ministry of Health, infected dogs were euthanized with intravenous injection of 19% potassium chloride (Darrow, Rio de Janeiro, Brazil), and specimens of popliteal lymph nodes and spleen were collected for immunohistochemistry. Lymph node and spleen specimens of dogs (n = 6) from a non-endemic VL region were used as negative controls. This study was approved by the Ethics Committee on Animal Experimentation and Animal Welfare, Faculty of Medicine, University of São Paulo, under protocol 296/10.

### Canine visceral leishmaniasis diagnosis

Dogs were subjected to serological diagnosis using an ELISA, as described previously [12]. Briefly, 96 flat-bottom microtiter wells were sensitized with 100 μL of crude total antigen, 10 μg/mL of *L. (L.) infantum chagasi* promastigotes protein in a carbonate–bicarbonate buffer (0.1 M pH 9.5), and incubated in a humidified chamber overnight at 4°C. After washing with a 0.15 M pH 7.2 phosphate buffer containing 0.05% Tween-20 (PBS-T), the plates were blocked with a solution of 10% skimmed milk powder in PBS-T incubated in a humid chamber for 2 h at 37°C. Following another wash, samples of the test sera, as well as the positive and negative controls, were added in duplicate at a dilution of 1:400 in PBS-T and incubated at 37°C for 1 h in a humidified chamber. The plates were washed three times with PBS-T, and anti-canine IgG conjugated to alkaline phosphatase (Bethyl Laboratories, Inc, Montgomery, TX, USA) was added at a 1:2000 dilution in PBS-T and incubated at 37°C for 45 min in a humidified chamber. The color reaction was performed with a chromogenic substrate (1.0 mg/mL pNPP; Sigma-Aldrich Corp, St. Louis, MO, USA) in a 0.1 M pH 9.5 carbonate–bicarbonate buffer and incubated at 25°C for 30 min. The reaction was stopped with 3 M NaOH, and the absorbance was read with a 405 nm filter. The cut-off of the reaction was calculated from the mean absorbance of the 20 sera of healthy uninfected dogs added to three standard deviations.

Parasitological diagnosis was based on the direct observation of *Leishmania* amastigote forms in popliteal lymph node smears obtained by needle aspiration biopsy and stained using a quick Romanovsky-type stain (Panótico Rápido®, Laborclin, Pinhais, Brazil). Lymph node biopsies were also used for parasite isolation using Schneider’s Drosophila medium (Sigma-Aldrich Corp.), supplemented with 10% heat-inactivated fetal bovine serum (FBS), 10 μg/mL of gentamicin, and 100 IU/mL of penicillin at 25°C. The isolated parasites were identified using monoclonal antibodies and isoenzyme electrophoretic profiles in the Laboratory of Leishmaniasis, Evandro Chagas Institute (Belém, Pará State, Brazil).

### Immunohistochemistry

#### *Leishmania* amastigotes

The immunohistochemical reaction for the detection of *Leishmania* amastigotes in the lymph nodes and spleen were developed according to Moreira et al. [13] using total hyperimmune serum of mice chronically infected with *L. (L.) amazonensis* as the primary antibody, and the LSAB kit (DakoCytomation, Carpintaria, CA, USA). As a positive control, histological sections of the spleen or lymph nodes of dogs with rare to moderate *Leishmania* parasitism were used, and the negative control was performed by omitting the primary antibody.

### Inducible nitric oxide synthase

Following deparaffinization, sections were hydrated and antigen retrieval was performed in 10 mM of citric acid solution (pH 6.0) in a water bath at 96 – 99°C. Endogenous peroxidase was blocked with 3% hydrogen peroxide and non-specific binding with 10% skimmed milk powder (Molico-Nestlé, São Paulo, SP, Brazil). Next, sections were incubated with the primary antibody (polyclonal rabbit anti-NOS2 [sc-651]; Santa Cruz Biotechnology, Inc, Dallas, TX, USA) diluted to 1:1000 in phosphate buffered saline (PBS) containing 1% bovine serum albumin (BSA) overnight at 4°C in a humidified chamber. To develop the reaction, the Novolink kit (Novocastra, Leica, Nussloch, Germany) was employed, followed by the chromogenic substrate DAB + H_2_O_2_ kit (DakoCytomation, Glostrup, Denmark). The sections were counter-stained with Harris hematoxylin, followed by dehydration, and the slides were mounted with a coverslip using resin. Histological sections of lymph nodes and spleens of dogs from non-endemic VL areas, with negative parasitological and serological diagnoses, were used as controls.

### Parasites and iNOS^+^ cell density

To evaluate the amastigotes and iNOS^+^ cell density, a quantitative morphometric analysis was performed. For this, ten fields for each histological section were photographed at a 40× objective, and the number of amastigotes or positive cells was quantified using the Axiovision 4.0 software on a computer coupled to a light microscope (Axioskop 2 plus; Carl-Zeiss, Jena, Germany). Subsequently, the density of immunostained cells and parasites was calculated considering the average number of parasites and cells per mm^2^.

### IFN-γ determination

IFN-γ levels in the sera of dogs naturally infected with *L. (L.) infantum chagasi* (n = 28) and control animals from a non-endemic VL area (n = 20) were determined with the commercial DuoSet ELISA kits (R&D Systems, Minneapolis, MN, USA), following the manufacturer’s instructions. Briefly, a flat-bottom microtiter plate with 96 wells was sensitized with the capture antibody and incubated overnight at 25°C. Following three washes with 0.05% PBS-T, blocking was performed with 1% BSA–PBS for 1 h incubation at 25°C. After washing, a standard curve was used, and sera were added and incubated for 2 h at 25°C. Following another wash, the plate was incubated with detection antibodies for 2 h at 25°C. After washing, streptavidin was added and incubated for 20 min at 25°C, followed by incubation with the substrate (TMB Substrate Reagent Set, BDBiosciences, San Diego, CA, USA) for 20 min more. The reaction was stopped with 2 N H_2_SO_4_ and absorbance was measured at 450 nm in an ELISA reader. The standard curve equation was determined, and the concentration of IFN-γ in samples was calculated.

### Statistical analysis

Statistical analysis was performed using the software GraphPad PRISM 5.0 for Windows (GraphPad Software Inc, La Jolla, CA, USA). The non-parametric Mann–Whitney test was used to compare the number of parasites and iNOS^+^ cells labeled by immunohistochemistry and the levels of IFN-γ among clinical groups. The Spearman test was used to evaluate the correlation among the variables, considering all infected (symptomatic and asymptomatic) animals. Differences were considered significant when the *P*-value was less than 0.05.

## Results and discussion

Considering that CVL is a severe systemic disease and that there are only a few, controversial reports discussing the role of NO metabolites for controlling macrophage intracellular parasitism, this study was conducted to evaluate the correlation between parasite burden and macrophagic cells expressing iNOS in lymph nodes and in spleens of dogs affected by VL.

According to clinical status and laboratory evaluation, the 28 infected dogs included in this study were divided into symptomatic and asymptomatic groups. Asymptomatic dogs did not present external clinical signs, and all showed total serum protein levels <8.5 mg/dL and serum creatinine levels within normal limits, according to the International Renal Interest Society [14]. The symptomatic group presented with clinical signs of leishmaniasis, such as lymphadenomegaly (89%), splenomegaly (83%), weight loss (78%), onycogryphosis (61%), hepatomegaly (56%), and skin lesions (50%); moreover, 53% presented with increased serum creatinine (>1.4 mg/dL) and 60% had hyperproteinemia (>9.0 g/dL). Among the 28 infected dogs, 14 were females and 14 were males. A total of 39% (11/28) were less than 2 years old, 46% (13/28) were between 2 and 6-years-old, and 15% (4/28) were more than 6-years-old. The animals showed homogeneous distribution with respect to gender and age between the clinical groups, and neither variable was correlated with clinical or immunohistochemical findings in this study (*P* > 0.05).

*L. (L.) infantum chagasi* infection led to diverse clinical and immunopathological features in dogs, depending on the infective parasite load, vector bite exposure, time of infection, nutrition, and genetic and immunological background of the host. Thus, transversal studies related to natural infection in dogs can generate controversial results, especially when animals are sorted in clinical groups, since asymptomatic clinical status can represent a resistance pole, but also an early phase of infection or a pre-patent disease period [15]. Two populations of asymptomatic dogs in endemic areas has been characterized: one seronegative, but with positive molecular detection of *L. (L.) infantum chagasi*, and another with positive serology and molecular results for the parasite, suggesting that asymptomatic dogs have a dichotomous infection spectrum that can influence humoral and cellular immunological status during CVL infection [16].

**Figure 1:**
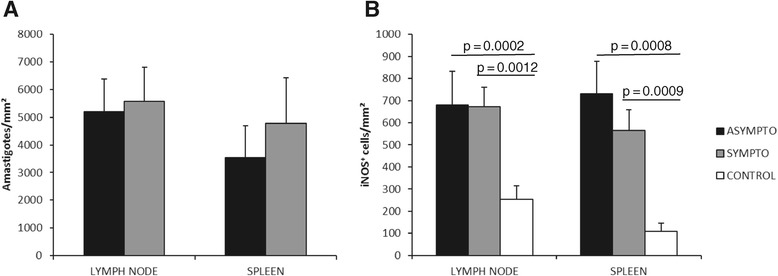
**Average and standard error of the density of parasites (A) and iNOS** ^**+**^ **cells (B) in the lymph nodes and spleen of asymptomatic and symptomatic dogs naturally infected with** ***Leishmania (Leishmania) infantum chagasi*** **, and control animals from a non-endemic visceral leishmaniasis area.** Data analysed by the Mann–Whitney test.

All dogs included in this study showed positive serology and amastigotes in popliteal lymph node smears. Parasites were isolated from six of the symptomatic and four of the asymptomatic dogs, and were characterized as *Leishmania (L.) infantum chagasi* by monoclonal antibodies and isoenzyme electrophoretic profiles. As it was not possible to isolate and characterize samples from all dogs used in this study, we could not exclude the possibility that some dogs were infected with other species of parasites. However, it is worth noting that despite a report of two dogs infected with *Leishmania (L.) amazonensis* in the municipality of Araçatuba, no cases of human disease nor the natural vector of this parasite species has been described in this region to date [17]. The immunohistochemical quantitative studies in lymph nodes or in spleens did not show significant differences (*P* = 0.2471 and *P* = 0.5158, respectively) between the clinical groups, supporting the idea that our infected asymptomatic dogs could be in the pre-patent period of infection, as reported by Coura-Vital et al. [16]. Figure 1A shows the number of parasites/mm^2^ in the popliteal lymph nodes and spleens of symptomatic and asymptomatic dogs. The density of parasites in popliteal lymph nodes in symptomatic dogs was 5,581 ± 1,232 amastigotes/mm^2^ and 5,201 ± 1,336 amastigotes/mm^2^ in the asymptomatic dogs. The density of parasites in the spleens in symptomatic dogs was 4,776 ± 904 amastigotes/mm^2^ and 3,533 ± 1,758 amastigotes/mm^2^ in asymptomatic dogs.

**Figure 2:**
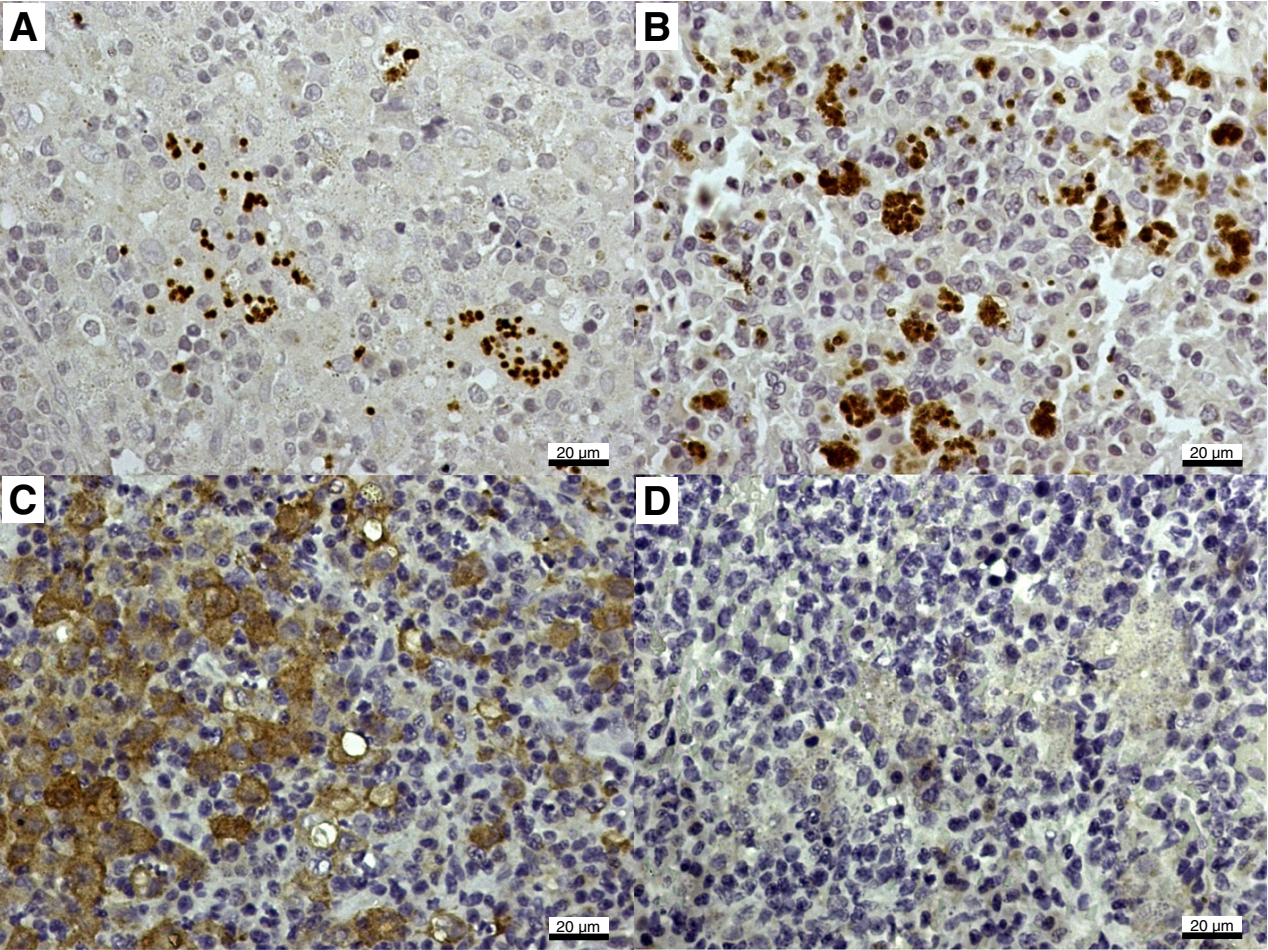
**Lymph node histological sections of asymptomatic (A, C) and symptomatic (B, D) dogs naturally infected with** ***Leishmania (Leishmania) infantum chagasi*** **showing amastigotes (A, B) and iNOS** ^**+**^ **cells (C, D) stained brown by immunohistochemistry.**

**Figure 3:**
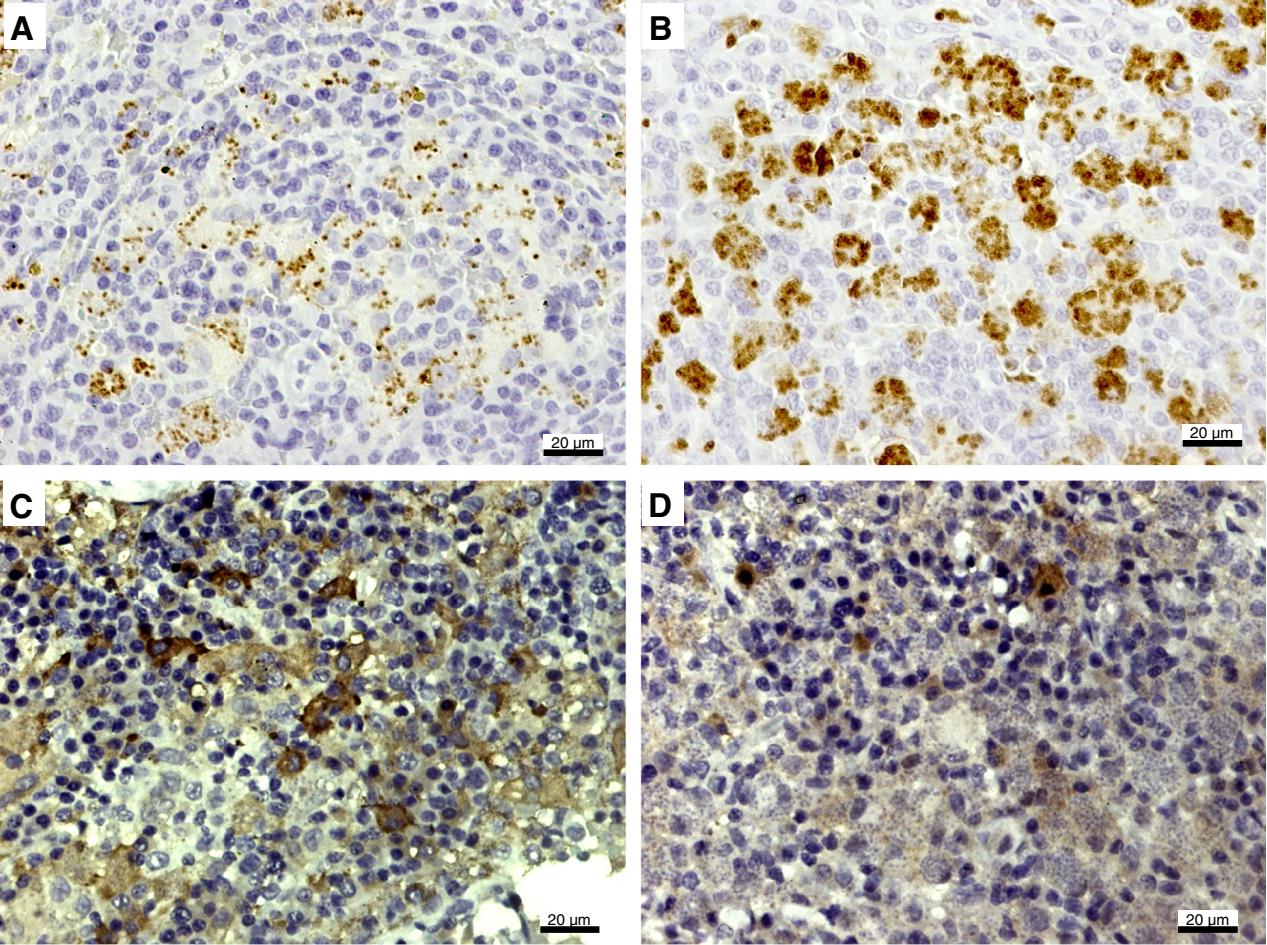
**Spleen histological sections of asymptomatic (A, C) and symptomatic (B, D) dogs naturally infected with** ***Leishmania (Leishmania) infantum chagasi*** **showing amastigotes (A, B) and iNOS** ^**+**^ **cells (C, D) stained brown by immunohistochemistry.**

It has been reported that the elimination of intracellular *Leishmania* amastigotes occurs through the production of oxygen [18] and nitrogen-reactive species [9,19]. The microbicidal production of NO requires iNOS action, which uses L-arginine as a substrate, converting it to L-citrulline and NO. The present study showed that dogs affected by VL presented with an increased expression of iNOS since both symptomatic and asymptomatic dogs had a higher number of iNOS^+^ cells than uninfected controls (*P* < 0.05), without a significant difference between the clinical groups (Figure 1B); the number of iNOS^+^ cells/mm^2^ in lymph nodes was 671 ± 100 and 681 ± 162 (*P* = 0.3257) in symptomatic and asymptomatic dogs, respectively, while in the spleen the numbers were 566 ± 107 and 731 ± 147, respectively (*P* = 0.5940). Figures 2 and 3 illustrate the immunohistochemistry reaction for parasites and iNOS detection in the popliteal lymph nodes and spleens of asymptomatic and symptomatic dogs.

The Spearman correlation test was applied considering symptomatic and asymptomatic dogs as one group of infected animals, since no difference was observed in the number of parasites and iNOS^+^ cells between them. A negative correlation was observed between the number of parasites and the number of iNOS^+^ cells in lymph nodes (*r* = −0.5341; *P* = 0.00341) and in the spleen (*r* = −0.4669; *P* = 0.0329) of infected dogs, suggesting that NO could be a factor involved in controlling parasitism in these tissues.

**Figure 4:**
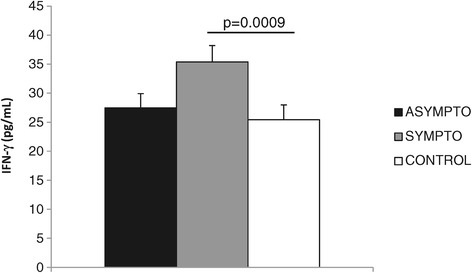
**Average and standard error of the concentration (pg/mL) of IFN-γ in the sera of asymptomatic and symptomatic dogs naturally infected with** ***Leishmania (Leishmania) infantum chagasi*** **, as well as control animals from a non-endemic visceral leishmaniasis area.** Data analysed by the Mann–Whitney test.

In vitro experiments have shown that canine macrophages pre-activated with rIFN-γ and infected with *L. (L.) infantum* produce higher levels of iNOS, which is accompanied by a decrease in the number of parasites in their cytoplasm when compared to macrophages without pre-activation, revealing the role of IFN-γ in iNOS induction [8]. Herein, the serum concentrations of IFN-γ were positively correlated with iNOS^+^ cell density in the lymph nodes of infected dogs (*r* = 0.3776; *P* = 0.0303), but not in the spleen. Moreover, the concentration (pg/mL) of IFN-γ was higher in infected symptomatic dogs compared to controls (*P* = 0.0009), but asymptomatic dogs did not have significant differences in IFN-γ between symptomatic (*P* = 0.6729) and control dogs (*P* = 0.8200). The average and standard error of IFN-γ concentration was 35.39 ± 2.82 pg/mL in symptomatic, 27.47 ± 2.46 pg/mL in asymptomatic, and 25.43 ± 2.55 pg/mL in control dogs (Figure 4). A recent report demonstrated mixed Th1 and Th2 cytokine gene expression profiles in the blood leucocytes of symptomatic dogs, but with dominant IFN-γ gene expression in the lymph nodes [20]. The enhanced accumulation of IFN-γ mRNA was also observed in the bone marrow of dogs infected with *L. (L.) infantum chagasi*, suggesting that the clinical symptoms are not due to a deficiency in IFN-γ production, but probably by detectable interleukin (IL)-4 mRNA, which is significantly higher in symptomatic dogs [21].

In addition, it has been shown that the expression of iNOS in the spleens of dogs naturally infected by *L. (L.) infantum chagasi* is associated with clinical manifestation and high parasite load [10]. Moreover, arginase activity has been detected in *Leishmania*. Since arginase and iNOS compete for the use of L-arginine as a substrate, the availability of this amino acid for both pathways is critical for parasite replication. So, the parasite must escape from several microbicidal mechanisms to survive inside macrophages of vertebrate hosts, such as NO production mediated by iNOS [22].

## Conclusion

The negative correlation observed between tissue parasitism and the expression of iNOS may be a reflection of NO acting on the control of intracellular amastigotes. However, additional studies should be conducted to assess the actual contribution of nitrogen, as well as reactive oxygen species, on the lysis of *Leishmania* amastigotes in canine infection.

## References

[1] Shaw JJ (2006). Further thoughts on the use of the name *Leishmania (Leishmania) infantum chagasi* for the aetiological agent of American visceral leishmaniasis. Mem Inst Oswaldo Cruz.

[2] Dantas Torres F (2007). The role of dogs as reservoirs of *Leishmania* parasites, with emphasis on *Leishmania (Leishmania) infantum* and *Leishmania (Viannia) braziliensis*. Vet Parasitol.

[3] Reis AB, Martins-Filho OA, Teixeira-Carvalho A, Carvalho MG, Mayrink W, Franca-Silva JC, Giunchetti RC, Genaro O, Corrêa-Oliveira R (2006). Parasite density and impaired biochemical/hematological status are associated with severe clinical aspects of canine visceral leishmaniasis. Res Vet Sci.

[4] Feitosa MM, Ikeda FA, Luvizotto MCR, Perri SHV (2000). Aspectos clínicos de cães com leishmaniose visceral no município de Araçatuba – São Paulo (Brasil). Clin Vet.

[5] Pinelli E, Killick-Kendrick R, Wagenaar J, Bernardina W, Del Real G, Ruitenberg EJ (1994). Cellular and humoral immune response in dogs experimentally and naturally infected with *Leishmania infantum*. Infec Immunity.

[6] Silveira FT, Carneiro LA, Ramos PK, Chagas EJ, Lima LV, Campos MB, Laurenti MD, Gomes CM, Corbett CE (2012). A cross-sectional on canine *Leishmania (L.) infantum chagasi* infection in Amazonian Brazil ratifies a higher prevalence of specific IgG-antibody response than delayed-type hypersensitivity in symptomatic and asymptomatic dogs. Parasitol Res.

[7] Pinelli E, Van der Kaaij SY, Slappendel R, Fragio C, Ruitemberg EJ, Bernardina W, Rutten VP (1999). Detection of canine cytokine gene expression by reverse transcription-polymerase chain reaction. Vet Immunol Immunopathol.

[8] Sisto M, Brandonisio O, Panaro MA, Acquafredda A, Leogrande D, Fasanella A, Trotta T, Fumarola L, Mitolo V (2001). Inducible nitric oxide synthase expression in *Leishmania*-infected dog macrophages. Comp Immunol Microbial Infec Dis.

[9] Zafra R, Jaber JR, Pérez-Écija RA, Barragán A, Martínez-Moreno A, Pérez J (2008). High iNOS expression in macrophages in canine leishmaniasis is associated with low intracellular parasite burden. Vet Immunol Immunopathol.

[10] Rocha dos Santos F, Vieira PMA, Corrêa-Oliveira R, Giunchetti RC, Carneiro CM, Reis AB, Malaquias LCC (2011). Qualitative and quantitative immunohistochemical evaluation of iNOS expression in the spleen of dogs naturally infected with *Leishmania chagasi*. Parasitol Res.

[11] Santos PL, Costa RV, Braz JM, Santos LFVC, Batista AC, Vasconcelos CRO, Rangel MR, Ribeiro de Jesus A, De Moura TR, Leopoldo PTG, Almeida RP (2012). *Leishmania chagasi* naturally resistant to nitric oxide isolated from humans and dogs with visceral leishmaniasis in Brazil. Nitric Oxide.

[12] Colombo FA, Odorizzi RM, Laurenti MD, Galati EA, Canavez F, Pereira-Chioccola VL (2011). Detection of *Leishmania (Leishmania) infantum* RNA in fleas and ticks collected from naturally infected dogs. Parasitol Res.

[13] Moreira MA, Luvizotto MC, Garcia JF, Corbett CEP, Laurenti MD (2007). Comparison of parasitological, immunological and molecular methods for the diagnosis of leishmaniasis in dogs with different clinical signs. Vet Parasitol.

[14] International Renal Interest Society: *IRIS Guidelines [Internet].* Basel: 2006. [cited 2012 Mar]. Available from: http://www.iris-kidney.com/guidelines/.

[15] Oliva G, Scalone A, Foglia Manzilli V, Gramiccia M, Pagano A, Di Muccio T, Gradoni L (2006). Incidence and time course of *Leishmania infantum* infections examined by parasitological, serological and nested-PCR techniques in a cohort of naive dogs exposed to three consecutive transmission seasons. J Clin Microbiol.

[16] Coura-Vital W, Marques MJ, Giunchetti RC, Teixeira-Carvalho A, Moreira ND, Vitoriano-Souza J, Vieira PM, Carneiro CM, Corrêa-Oliveira R, Martins-Filho OA, Carneiro M, Reis AB (2011). Humoral and cellular immune response in dogs with inapparent natural *Leishmania* infantum infection. Vet J.

[17] Tolezano JE, Uliana SR, Taniguchi HH, Araújo MF, Barbosa JA, Barbosa JE, Floeter-Winter LM, Shaw JJ (2007). The first records of *Leishmania (Leishmania) amazonensis* in dogs (*Canis familiaris*) diagnosed clinically as having canine visceral leishmaniasis from Araçatuba county, São Paulo state, Brazil. Vet Parasitol.

[18] Pearson RD, Sullivan JA, Roberts D, Romito R, Mandell GL (1983). Interaction of *Leishmania donovani* promastigotes with human phagocytes. Infect Immunol.

[19] Liew FY, Millot S, Parkinson C, Palmer RMJ (1990). Macrophage killing of *Leishmania* parasites in vivo is mediated by nitric oxide from L-arginase. J Immunol.

[20] Barbosa MA, Alexandre-Pires G, Soares-Clemente M, Marques C, Rodrigues OR, De Brito TV, da Fonseca IP, Alves LC, Santos-Gomes GM (2011). Cytokine gene expression in the tissues of dogs infected by *Leishmania infantum*. J Comp Pathol.

[21] Quinnell RJ, Coutenay O, Shaw MA, Day MJ, Garcez LM, Dye C, Kaye PM (2011). Tissue cytokine responses in canine visceral leishmaniasis. J Infec Dis.

[22] Da Silva MF, Floeter-Winter LM (2014). Arginase in *Leishmania*. Subcell Biochem.

